# Parkinson’s disease: Individual placebo responses are predicted by baseline expression of a striato-limbic network

**DOI:** 10.21203/rs.3.rs-10119103/v1

**Published:** 2026-06-24

**Authors:** János Barbero, An Vo, Joshua J. Strohl, Sebastiano Vacca, Chris C. Tang, Nha Nguyen, Prashin Unadkat, Yilong Ma, Shichun Peng, Martin Niethammer, Brage Brakedal, Johan Wallin, Ioanna Markaki, Per Svenningsson, Michael G. Kaplitt, Charalampos Tzoulis, David Eidelberg

**Affiliations:** Center for Neurosciences, The Feinstein Institutes for Medical Research, Manhasset, NY, USA; Center for Neurosciences, The Feinstein Institutes for Medical Research, Manhasset, NY, USA; Center for Neurosciences, The Feinstein Institutes for Medical Research, Manhasset, NY, USA; Center for Neurosciences, The Feinstein Institutes for Medical Research, Manhasset, NY, USA; Center for Neurosciences, The Feinstein Institutes for Medical Research, Manhasset, NY, USA; Center for Neurosciences, The Feinstein Institutes for Medical Research, Manhasset, NY, USA; Center for Neurosciences, The Feinstein Institutes for Medical Research, Manhasset, NY, USA; Center for Neurosciences, The Feinstein Institutes for Medical Research, Manhasset, NY, USA; Center for Neurosciences, The Feinstein Institutes for Medical Research, Manhasset, NY, USA; Center for Neurosciences, The Feinstein Institutes for Medical Research, Manhasset, NY, USA; Neuro-SysMed, Department of Neurology, Haukeland University Hospital, Bergen, Norway; Department of Clinical Neuroscience, Karolinska Institutet, Stockholm, Sweden; Department of Clinical Neuroscience, Karolinska Institutet, Stockholm, Sweden; Department of Clinical Neuroscience, Karolinska Institutet, Stockholm, Sweden; Department of Neurological Surgery, Weill Cornell Medical College, New York, NY, USA; Neuro-SysMed, Department of Neurology, Haukeland University Hospital, Bergen, Norway; Center for Neurosciences, The Feinstein Institutes for Medical Research, Manhasset, NY, USA

## Abstract

Parkinson’s disease (PD) is associated with substantial placebo effects. We used network analysis to identify a specific sham surgery-related pattern (SSRP) in metabolic PET data from a double-blind PD gene therapy trial. Baseline SSRP expression measured before randomization correlated with motor improvement under the blind after sham surgery. To validate this predictive relationship, we measured baseline SSRP levels in two independent placebo-controlled trials of pharmacologic PD treatments administered orally or by subcutaneous injection. As with sham surgery, pre-randomization SSRP expression correlated with placebo responses in each of the validation groups. Using magnetic resonance diffusion tensor imaging (DTI), we found that individual differences in SSRP expression and placebo response were attributable to variation in the density of fiber tracts linking key network nodes, particularly the nucleus accumbens and the anterior cingulate cortex. The findings support SSRP as a network-based imaging marker of placebo susceptibility in PD clinical trials.

## Teaser

Placebo responses in Parkinson’s disease clinical trials are predicted by baseline expression of a specific brain network.

## INTRODUCTION

Placebo effects represent a significant confound in randomized clinical trials (RCTs) for Parkinson’s disease (PD), posing a major challenge in blinded efficacy studies of new potential treatments for the disorder (1). Indeed, approximately 16% of PD participants who receive oral placebo in pharmacological RCTs of this sort exhibit sustained improvement in motor ratings under the blind – a figure that increases to 42% in blinded surgical trials (2–5). Previous research has demonstrated that placebo responses in PD involve dopamine release in the ventral striatum, which can be influenced by prior treatment with dopaminergic medication and by the expectation of clinical benefit (6–8). However, the specific neural circuitry that mediates these effects is unknown.

In earlier work, we used metabolic positron emission tomography (PET) and network analysis to identify a unique spatial covariance topography in PD patients undergoing sham surgery (bilateral burr hole placement) as part of a double-blind gene therapy trial (9–11). This network, known as the sham surgery-related pattern (SSRP) (Fig. S1), was characterized by relative metabolic increases in the ventral striatum, anterior cingulate cortex and subcallosal region, as well as the hippocampus, amygdala, and posterior cerebellar vermis (9). Expression levels (subject scores) for this network increased consistently under the blind during the 6 months following the sham procedure. Importantly, the computational algorithm used to identify the SSRP relied on the presence or absence of a consistent monotonic trend in pattern expression over time, rather than individual changes in motor ratings recorded under the blind (9, 10, 12, 13). That said, clinical responses to sham surgery correlated with concurrent changes in SSRP expression, and surprisingly also with network levels determined in baseline scans obtained before randomization (9, 10). Given the excellent test-retest reproducibility of SSRP expression and its stability of measure in the face of dopaminergic treatment and disease progression (9), we wished to explore the potential utility of the baseline measure as a predictor of the placebo response in individual PD trial participants.

Before doing so, however, several critical issues needed to be addressed. For one thing, as an imaging marker of the PD placebo response, predictions of blinded motor outcomes should be independent of the mode of administration, i.e., whether the placebo is given pharmacologically as an oral pill or as a subcutaneous injection, or surgically through a sham procedure. Likewise, it is important to know the extent to which subject variables such as age, sex, disease duration and severity, and medication status influence the placebo response in this population (4, 5). By the same token, genes that regulate brain dopamine concentrations, such as the catechol-O-methyltransferase (*COMT*) rs4680 single nucleotide polymorphism also may influence the placebo response (14).

Here, we show that despite marked differences in trial design, frequency and route of administration, disease severity, and medication status, motor outcomes under the blind correlated with baseline SSRP expression in three independent PD trials (see Methods). Moreover, the topological response of the SSRP to placebo was stereotyped in the three cohorts so as to maximize information flow through the network. Finally, we show how active treatment can sustain the changes in network organization that were induced by placebo.

## RESULTS

### SSRP as a network marker of the motor response to sham surgery in PD patients

**Figure 1:**
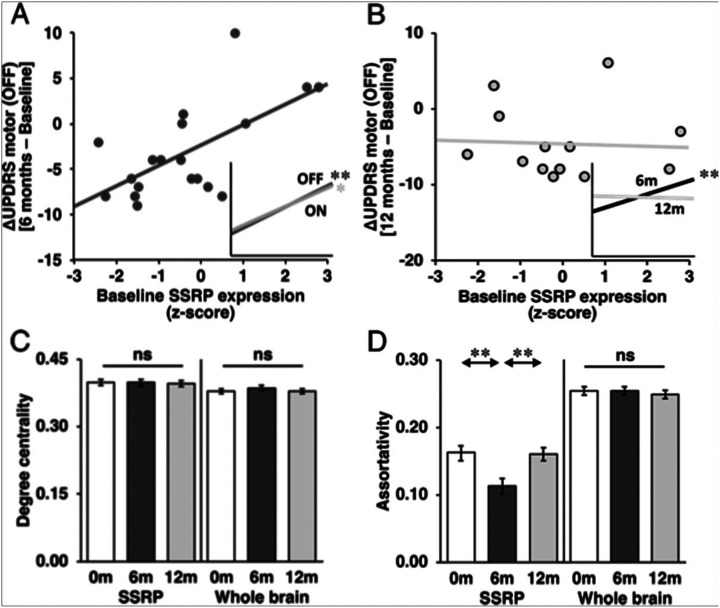
Motor responses to sham surgery correlate with baseline SSRP expression: Reversible network reorganization under the blind. **(A)** Plot showing a significant correlation between baseline SSRP expression and the change from baseline in off-state (OFF) motor ratings (ΔUPDRS) at 6 months (r=0.63, p=0.004; Pearson correlation) in 19 PD patients who were randomized to sham surgery (PLs) in a double-blind gene therapy trial (see Methods). *Inset*: A similar correlation with baseline SSRP was seen for on-state (ON) ΔUPDRS motor ratings (r=0.51, p<0.03) (Table S2A). The ranges of the variables in the inset are the same as in the main panel. **(B)** Absence of correlation between baseline SSRP expression and off-state ΔUPDRS at 12 months (r=−0.05, p=0.87) in the 13 PLs subjects who were scanned before and after unblinding (see text). *Inset*: Comparison with the significant correlation between these measures seen under the blind at 6 months in the same subject (see text). **(C)** Bar graphs (mean ± SE) showing that degree centrality for the SSRP network (*left*) and for the whole brain (*right*) were unchanged under the blind and after unblinding (p>0.58; one-way ANOVA). **(D)** By contrast, SSRP assortativity decreased from baseline to 6 months (−30%, p<0.01; Tukey-Kramer HSD). These changes were reversed by unblinding, as shown by a significant increase to baseline levels from 6 to 12 months (+41.4%, p<0.01). Changes in whole brain assortativity over time were not significant (p=0.74; one-way ANOVA). (See Table 1 for summary of changes in graph metrics for the SSRP and the other networks and for the whole brain.) [*p<0.05, **p<0.01, ***p<0.001]

The clinical responses to placebo under the blind are presented for the PLs, PLi, and PLo groups in Table S1A-C. Baseline SSRP expression levels computed pre-randomization in the PLs subjects (n = 19) correlated with improvement in on- and off-state motor ratings under the blind 6 months after sham surgery (OFF: r = 0.63, p < 0.005; ON: r = 0.51, p = 0.03; Pearson correlations) (Fig. 1A). Indeed, the PLs subjects with the lowest network expression at baseline exhibited the largest improvement in UPDRS motor ratings under the blind in both medication states. The linear regression model to predict off-state placebo motor responses was not improved by the addition of baseline UPDRS motor ratings as a covariate. Of these subjects, 13 subjects were unblinded between 6 and 12 months, such that the final imaging session was conducted in the “open-label” condition (see Methods). Baseline SSRP expression in these subjects did not correlate with unblinded motor responses at 12 months, whether measured onor off-dopaminergic medication (OFF: r=−0.09, p = 0.78; ON: r = 0.2, p = 0.53) (Fig. 1B, Table S2A). The correlation between the blinded motor response to sham surgery and baseline network expression was specific for the SSRP: significant predictive relationships were not observed for the PDRP, a well-characterized motor-related metabolic network associated with PD or for the normal default mode network (DMN) (Table S2B, C) (see Methods).

We next used graph analysis to examine the effects of blinding and unblinding on SSRP functional organization in the PLs group. Distinctive changes in graph metrics were observed under the blind following sham surgery (Table 1). Degree centrality (Fig. 1C), a measure of network connectivity, was unchanged under the blind at 6 months, both in the SSRP space (0.0%, p = 1.0; Tukey-Kramer HSD) and over the brain as a whole compared to baseline (+ 1.7%, p = 0.66). By contrast, assortativity (Fig. 1D), a measure of the homogeneity of node-to-node connections across a graph, showed significant reductions under the blind for the SSRP space (−30.0%, p < 0.005).

The network-specific changes in SSRP assortativity suggest a shift toward more efficient information flow through the network during expectation. Surprisingly, this functional change was reversed by unblinding, with return of SSRP assortativity to baseline levels at 12 months (+ 41.4%, p = 0.005 compared to 6 months; Tukey-Kramer HSD). That said, analogous assortativity changes for the whole brain were not seen when comparing the blinded 6-month time point to baseline (+ 0.0%, p = 1.0), or to the unblinded 12-month time point (−2.3%, p = 0.77). Likewise, no significant changes in PDRP degree centrality (−1.4%, p = 0.72) and assortativity (+ 4.7%, p = 0.70) were seen under the blind at 6 months compared to baseline. However, a significant reduction in PDRP degree centrality was seen at 12 months (−8.3%, p < 0.0001) without a significant change in assortativity (+ 8.5%, p = 0.28) over the same time interval. This contrasted with DMN assortativity which declined under the blind at 6 months relative to baseline (−24.0%, p < 0.0001; Student’s *t*-test) and was partially restored by unblinding (+ 14.1%, p = 0.047). Apart from the changes in degree centrality and assortativity, only minimal inconsistent changes in the other graph metrics were observed under the blind for the SSRP and the other pre-specified networks that were computed in the PLs data (Table 1).

### SSRP and the long-term placebo response in early-stage levodopa-treated PD patients

**Figure 2:**
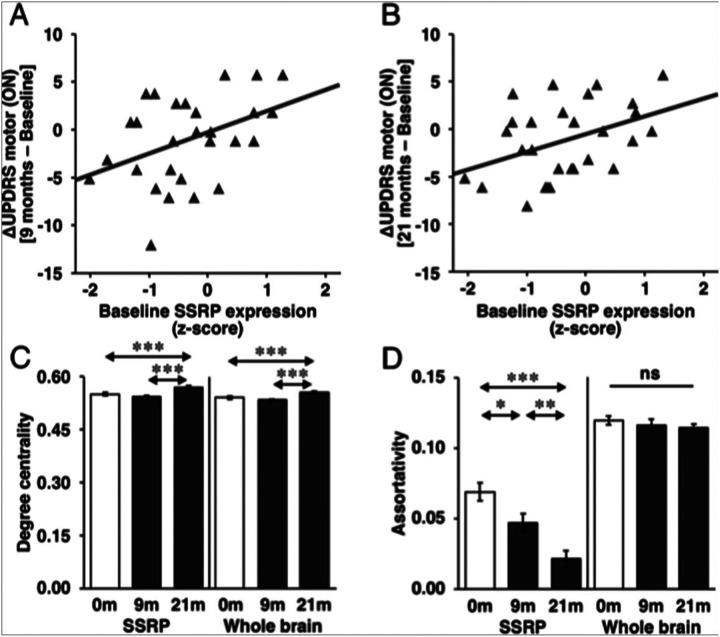
Long-term prediction of placebo motor responses: Sustained network reorganization under the blind. **(A, B)**Plots showing significant correlations between baseline SSRP expression and ΔUPDRS motor ratings in the 28 PD subjects who were randomized to subcutaneously injected placebo (PLi) in a double-blind disease modification trial (see Methods). Significant predictive relationships with baseline SSRP were seen for on-state (ON) motor responses at both blinded time points (9 months: r=0.42, p=0.028; 12 months: r=0.41, p=0.030). These correlations, however, were not significant for off-state (OFF) ΔUPDRS motor ratings obtained at either time point (p>0.10) (Table S2A). **(C)** Degree centrality for the SSRP network was unchanged with respect to baseline at 9 months (−1.4%, p=0.17; Tukey-Kramer HSD), but increased significantly at 21 months with respect to each of the prior time points (p<0.01). A similar increase in degree centrality was likewise observed for the whole brain at 21 months (p<0.01) (see Table 1). **(D)** By contrast, a stepwise decline in SSRP assortativity was observed over time in this cohort with significant reductions under the blind at 9 months (−31.8%, p=0.03), which declined to yet lower levels at 21 months with respect to baseline (−69.2%, p<0.0001). Whole brain assortativity in this cohort did not change over the same time interval (p=0.52; one-way ANOVA). [*p<0.05, **p<0.01, ***p<0.001]

Similar SSRP effects were observed in an independent group of PD patients who received weekly subcutaneous placebo injection (PLi) under a 21-month blind as part of a subsequent RCT. As in the PLs group at 6 months, we observed a significant correlation between baseline SSRP values and motor responses under the blind at 9 months (r = 0.42 p = 0.028), which was also present at 21 months (r = 0.41 p = 0.03; Pearson correlations) (Fig. 2A, B). While in PLs the correlation between baseline SSRP expression and the blinded motor response was significant in both the on- and off-dopaminergic medication states (Fig. 1A, *inset*), in PLi this relationship was present only in the on state (Table S2A). By the same token, the correlation of baseline SSRP expression with placebo motor responses in the on-state was improved by the addition of the *COMT* rs4680 polymorphism as a covariate (R^2^ = 0.34, p = 0.006; multiple linear regression). Of note, the *COMT* genotype did not contribute to the off-state regression model (see Methods), and baseline UPDRS motor ratings did not contribute to the prediction of motor responses under the blind in either medication state.

Graph analysis of the PLi data under the blind revealed changes in SSRP graph metrics similar to those observed for PLs. Significant changes in degree centrality (Fig. 2C) were not observed for the SSRP or for the whole brain at 9 months, although small increases in this metric were observed in both spaces at 21 months (SSRP: +5.0%, p < 0.0001; whole brain: +4.1%, p < 0.0005 compared to 9 months; Tukey-Kramer HSD) (Table 1). As in PLs, the time course of SSRP assortativity in PLi differed from degree centrality in that the metric declined under the blind at 9 months (−31.8%, p = 0.034), with further reductions as the blind phase continued from 9 to 21 months (−54.8%, p < 0.01) (Fig. 2D, *left*). Of note, assortativity measured across the whole brain did not change over time (p = 0.74) (Fig. 2D, *right*), nor were significant changes observed in the PDRP or DMN spaces (p > 0.15; one-way ANOVA, for both networks). Along these lines, significant changes in the other SSRP metrics were not observed at 9 months. Although reductions in clustering (−4.5%, p < 0.0001) and small-worldness (−2.7% p < 0.0005 compared to baseline) were present at 21 months, consistent changes in these metrics were not observed under the blind for the other two networks (Table 1).

### SSRP and the short-term placebo response in de novo Parkinson’s disease

**Figure 3:**
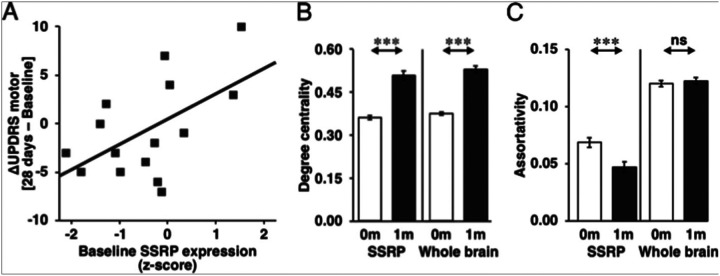
Baseline SSRP expression as a predictor and the role of network reorganization in the short-term placebo response. **(A)** Plots showing the correlation between baseline SSRP expression and the change in motor ratings (ΔUPDRS) under the blind in 15 unmedicated newly diagnosed subjects who were randomized to oral placebo in a pilot study of a potential disease modifying agent (see Methods). A significant correlation between these measures was observed under the blind at 28 days (r=0.55, p=0.034; Pearson correlation) (see Table S2A). **(B)** Degree centrality increased under the blind for the SSRP (+40.7%, p<0.0001) and the whole brain (+41.3%, p<0.0001; Student’s *t*-tests). **(C)** These changes contrasted with assortativity, which decreased from baseline to 28 days for SSRP (−31.5%, p<0.0001) while remaining unchanged for the whole brain (+1.8%, p=0.57).

We additionally explored the relationship between baseline SSRP expression and the response to orally administered placebo (PLo) given as part of a 30-day double-blind Phase 1 clinical trial in treatmentnaïve PD patients (15) (see Methods). Indeed, as in PLs and PLi, a significant correlation between these measures was also observed in the PLo subjects (r = 0.55, p = 0.03; Pearson correlation) (Fig. 3A). Other covariates such as the *COMT* rs4680 polymorphism or baseline motor ratings did not improve the accuracy of the SSRP model. Likewise, the changes in SSRP graph metrics observed under the blind that were similar to those in the other placebo groups (Table 1). Thus, in PLo, degree centrality (Fig. 3B) increased significantly under the blind in SSRP (+ 40.7% p < 0.0001; Student’s *t*-test) (*left panel*) and in the brain as a whole (+ 41.3%, p < 0.0001) (*right panel*). Notably, increases in degree centrality were also present in PDRP and DMN (PDRP: +31.4%; DMN: +16.2%, p < 0.0001 for both networks). That said, the changes in assortativity (Fig. 3C) seen concurrently in this group were highly network specific. As in the other placebo groups, this metric declined under the blind in PLo – but only in SSRP (−31.5%, p < 0.0001) (*left panel*) with no concurrent change over the whole brain (+ 1.8%, p = 0.57) (*right panel*). By contrast, significant *increases* in assortativity were observed in PDRP and DMN (+ 18.3% and + 36.9%, p < 0.001 for both networks) in response to short-term placebo (Table 1).

In summary, reduction in SSRP assortativity was a consistent feature of the placebo response, irrespective of the mode of delivery and the length of time spent under the blind. Despite the lower level of expectation associated with oral placebo and the relatively short blind phase in PLo, the decline in SSRP assortativity in this group (~ 30% with respect to baseline) was similar to that observed in the other groups. The PLo group additionally exhibited non-specific increases in degree centrality involving the whole brain, with parallel increases in both degree centrality and assortativity in the PDRP and DMN. Although changes in other graph metrics were also seen under the blind in each of the placebo groups, none favored a specific network. For example, both the clustering coefficient and small-worldness declined under the blind in PLo, but these changes involved all three networks as well as the whole brain. No significant relationship was detected between SSRP expression and the *COMT* rs4680 polymorphism in any of the placebo groups, individually or in combination (p > 0.6; Kendall Tau b correlation).

### Microstructural differences in SSRP pathways underlie susceptibility to placebo effects

**Figure 4:**
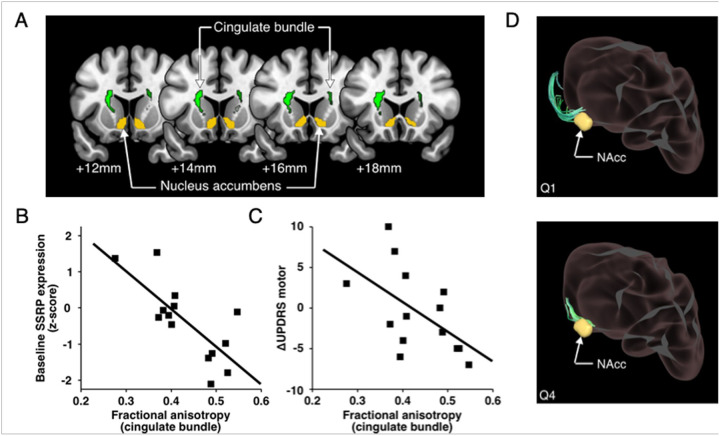
Baseline SSRP expression reflects individual differences in tracts connecting the anterior cingulate cortex to the nucleus accumbens. **(A)** Results of voxel-wise analysis of baseline MRI diffusion tensor imaging (DTI) scans. Using DTI scans acquired simultaneously with metabolic PET, we identified significant white matter clusters in which fractional anisotropy (FA), a measure of local microstructural integrity, correlated with pre-randomization SSRP expression measured in the same subjects (see Methods). These regions were localized bilaterally to the cingulate bundle (*green*) in the vicinity of the nucleus accumbens (*yellow*). **(B)** Plot of correlation between FA values for the regions displayed in **(A)** and corresponding baseline SSRP values measured in the PLo group (n=15). A significant inverse correlation was observed between these variables (r=−0.75, p<0.002; Pearson correlation). **(C)**Similarly, baseline FA values in these subjects correlated with motor improvement under the blind at 28 days (r=−0.55, p=0.041). **(D)**Reconstructions of fiber pathways linking the pregenual anterior cingulate cortex (pgACC) to the nucleus accumbens (NAcc) (see Methods). The upper panel shows the integrity of these tracts in a PLo subject from the bottom SSRP quartile (Q1) in whom motor ratings improved under the blind. The lower panel shows relatively reduced tract integrity in a PLo subject from the top SSRP quartile (Q4). In contrast to the first subject, motor ratings under the blind did not improve in this individual.

We used magnetic resonance diffusion tensor imaging (DTI) scans acquired simultaneously with FDG PET in the PLo subjects to explore the relationship of baseline SSRP expression and individual differences in the microstructural integrity of white matter tracts connecting key network nodes. This was done using voxel-wise regression of SSRP subject scores measured with FDG PET on maps of fractional anisotropy (FA), a measure of axonal integrity and coherence, obtained using DTI (see Methods). We used pre-randomization MRI/PET data from the 30 PD participants in the NADPARK trial (see Methods) to identify significant white matter regions in which local FA values correlated with SSRP measurements from the same subjects. Significant clusters (p < 0.05 corrected; SPM) were identified along the cingulate bundle (Fig. 4A, *green*) in proximity to the nucleus accumbens (NAcc, *yellow*), showing an inverse relationship between the two imaging measures (Fig. 4B). To demonstrate the stability of this finding, we assessed the test-retest reliability of FA values in this cluster by comparing measurements obtained at baseline and 28 days in each of the trial participants. Indeed, there was excellent agreement between cluster FA values at both time points across the group as a whole (ICC = 0.97) and in each of the trial arms (PLo: ICC = 0.95, Active drug: ICC = 0.98; intra-class correlation coefficients, p < 0.0001). As with SSRP expression, baseline FA values in the cingulate cluster correlated with changes in motor ratings under the blind in the PLo sample (r=−0.55 p = 0.041) (Fig. 4C).

In aggregate, the DTI data relate the strongest placebo responses in PLo to low baseline SSRP expression and relatively preserved cingulate bundle microstructure. By the same token, PLo subjects with weaker responses were distinguished by higher baseline SSRP expression and lower microstructural integrity in cingulate white matter. To visualize these differences, we used diffusion tractography to reconstruct the fiber pathways connecting NAcc with the pregenual anterior cingulate cortex (pgACC) in subjects with high or low baseline SSRP expression (see Methods). To this end, we stratified baseline DTI scans from all 30 trial participants by SSRP quartile and measured tract-specific FA along this projection in each subject (see Methods). Significant differences in tract-specific FA were seen across SSRP expression quartiles (η^2^ = 0.339, p = 0.018; one-way ANOVA), with lower tract integrity in the highest quartile and greater tract integrity in the lowest (p = 0.016; Tukey-Kramer HSD). Tract reconstructions are displayed for representative PLo subjects with strong and weak placebo responses, respectively (Fig. 4D, *top* and *bottom panels*). Although only approximately 25% of the placebo recipients in this study had DTI scans, the PLo data suggest that motor responses under the blind are determined, at least in part, by baseline connectivity differences in pathways linking NAcc and the anterior cingulate cortex.

### Network-based estimates of the placebo response in treated patients

**Figure 5:**
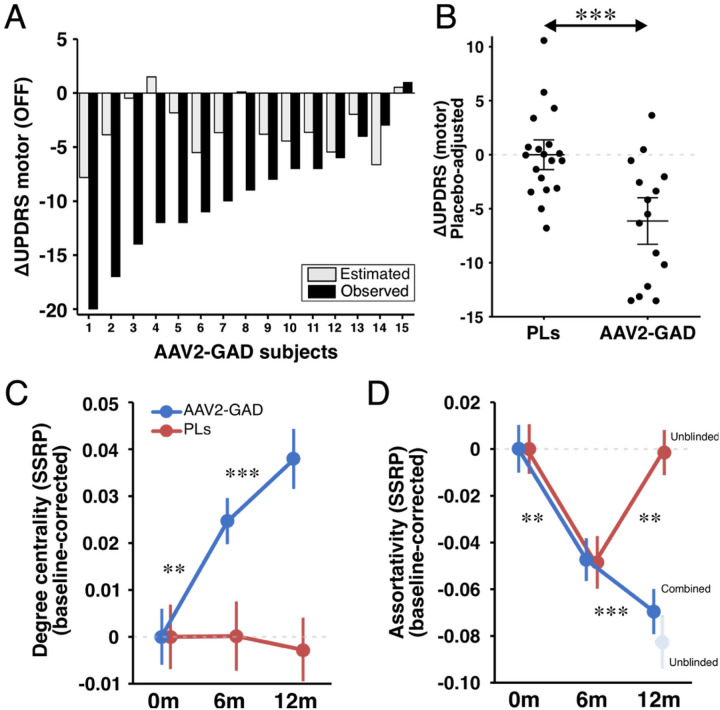
Estimating the latent placebo response in trial subjects randomized to active treatment. **(A)** Bar graph showing the change from baseline in off-state UPDRS motor ratings (ΔUPDRS (motor)) under the blind 6 months after subthalamic AAV2-GAD gene therapy (*black bars*). The linear relationship between severity-adjusted baseline SSRP expression and motor outcome in the PLs group was used to estimate the latent placebo response in each of the gene therapy subjects (*gray bars*) (see Methods). The observed response exceeded the estimated placebo response in 80% of the subjects, which was highlysignificant for the gene therapy group as a whole (p=0.00076; paired Student’s *t*-test). **(B)** Scatter diagram of placebo-adjusted changes in UPDRS motor ratings in individual subjects in the PLs and AAV2-GAD subjects. Gene therapy subjects showed greater placebo-adjusted improvement (see Methods) compared to their sham surgery counterparts (p=0.0007, Student’s *t*-test). [The mean±SE is displayed for each group.] **(C)** Time course of SSRP degree centrality in trial subjects receiving subthalamic AAV2-GAD gene therapy (*blue*) and PLs (*red*) (see text). SSRP degree centrality rose over time following gene therapy, with significant increases from baseline to 6 months (+7.2%, p=0.008) that persisted at 12 months (6m to 12m: +3.6%, p=0.24; 0m to 12m: +11.0%, p<0.0001). In PLs, by contrast, changes in SSRP degree centrality were not significant (p=0.94; one-way ANOVA). **(D)** Assortativity, however, declined under the blind after gene therapy (0m to 6m: −21.6%, p=0.002) that paralleled the corresponding PLs change (Fig. 1D). Nonetheless, rather than return to baseline after unblinding, SSRP assortativity in the AAV2-GAD group continued to decline (0m to 12m: −31.7%, p<0.0001). Reduction in this graph metric was significant (p<0.0001) even when consideration was limited to those gene therapy subjects (n=6) who were unblinded before the 12m time point (*light blue*; see text). [Each disc represents the mean±SE of the AUC for each SSRP metric, averaged over 100 bootstrap iterations at each time point (see Methods). The baseline mean for each of the metrics was subtracted from the corresponding values measured at the subsequent time points.]

Using regression analysis, we identified the best fit line that related baseline SSRP expression to the changes in UPDRS motor ratings under the blind in the PLs cohort (see above). This model was then applied to the subjects (n = 15) who received subthalamic (AAV2-GAD) gene therapy as previously reported (10, 16, 17) original subjects was considered to be an outlier with regard to the observed treatment response and was therefore not included in the subsequent analysis (see Methods). Pre-randomization SSRP expression values were used to estimate the latent placebo response for each of these trial participants. The resulting estimate (ΔUPDRS_EST_) was then compared to the observed treatment response (ΔUPDRS_OBS_) under the blind in the same individual. We found that the observed improvement in motor ratings at 6 months was greater than the estimated placebo response in 12 of the 15 gene therapy subjects (80.0%), similar in two of the subjects (13.3%), and lower in the remaining one subject (6.7%). On average, the observed motor outcome following gene therapy exceeded the estimated sham response in the same individuals (p < 0.001; paired Student’s t-test) (Fig. 5A). Likewise, subtracting ΔUPDRS_EST_ from ΔUPDRS_OBS_ in each subject yields the placebo-corrected ΔUPDRS, a measure of the individual change in motor ratings beyond the placebo effect. Comparing the distribution of these values in the gene therapy and sham surgery groups revealed significantly greater placebo-adjusted motor improvement in the treatment group (p < 0.001, Student’s *t*-test) (Fig. 5B).

Next, we tracked the time course of SSRP degree centrality and assortativity for the AAV2-GAD (n = 15) and PLs (n = 13) groups over the 12 months following surgery. Following gene therapy (*blue lines*), SSRP degree centrality (Fig. 5C) increased from baseline to 6 months (+ 7.2%, p = 0.008; Tukey-Kramer HSD), and remained elevated at 12 months (6m to 12m: +3.6%, p = 0.24; 0m to 12m: +11.0%, p < 0.0001). This contrasted with PLs (*red lines*) in which this SSRP metric did not change from baseline over the first postoperative year. By the same token, SSRP assortativity (Fig. 5D) declined under the blind in the AAV2-GAD group between baseline and 6 months (−21.6%, p = 0.0018) – a reduction commensurate with PLs over the same interval. Whereas in PLs, SSRP assortativity returned to baseline after unblinding, decline in this metric continued until 12 months in the gene therapy group (0m to 12m: −31.7%, p < 0.0001). Indeed, a similar reduction in SSRP assortativity was evident in the six AAV2-GAD subjects who were unblinded before the 12m imaging time point (p < 0.0001). Whole brain degree centrality increased from 0m to 6m after gene therapy (+ 6.5%, p = 0.0010), and continued to increase to 12m (6 to 12m: +7.1%, p = 0.0001; 0 to 12m: +14%, p < 0.0001). Whole brain assortativity remained unchanged, however, in both groups over the same time interval (p > 0.22, one-way ANOVA). Overall, the findings show that SSRP connectivity increases steadily over time after gene therapy but not sham surgery. SSRP assortativity, by contrast, exhibited comparable reductions under the blind in both groups – a topological response that was reversed by unblinding in the sham surgery group but which persisted after gene therapy.

## DISCUSSION

### The SSRP network and the placebo response

This study provides support for the role of the SSRP in mediating the placebo response in PD. While consistent increases in SSRP expression were seen under the blind in clinical responders to sham surgery (9), significant changes were not detected with oral or injected placebo. The absence of discernible activation of the SSRP in response to non-surgical placebo likely relates to the lower level of expectation associated with pharmacological versus surgical placebo, particularly in individuals with less severe disease (1, 3–5). Despite differences in the magnitude of the placebo response, the three groups exhibited stereotyped changes in network organization under the blind. Indeed, the topological placebo response, represented by consistent reduction in graph assortativity, was specific for the SSRP; placebo-induced changes of this sort were not observed for PDRP or DMN, or for the brain as a whole. Shifts in network configuration from baseline to low assortativity have been associated with increased robustness and reduced susceptibility to random node failure (18–22), as well as enhanced controllability through more efficient distribution of hub signals to peripheral low degree nodes (23). Interestingly, analogous topological shifts have been noted in healthy individuals performing cognitively demanding tasks (24–26). Although the network-level cognitive responses are typically recorded over hours rather than over weeks to months, in both scenarios functional connectivity was reorganized to maximize information flow through relevant networks. It is tempting to speculate that the topological response to placebo in the SSRP parallels the changes in network configuration observed during the performance of cognitive tasks. In particular, learning-related networks involving the dorsolateral prefrontal cortex (dlPFC) are also modulated by dopamine (27) and can be reconfigured by catecholamine release (28). Analogous topological changes involving the ventral medial prefrontal cortex (vmPFC) and anterior cingulate cortex (ACC) may underlie the placebo response through NAcc dopamine release and modulation of associated limbic pathways, with concomitant optimization of information flow through the SSRP network.

The specificity of the placebo-induced assortativity reductions for the SSRP was demonstrated by the absence of parallel topological changes in other relevant networks such as the PDRP. We note, however, that significant reduction in DMN assortativity was observed under the blind in the PLs group. This may relate to spatial overlap of the SSRP and DMN topographies in the ventral medial prefrontal and posterior cingulate regions (9, 29), and to placebo-mediated changes in functional connectivity between DMN nodes (30, 31). That said, the changes in DMN assortativity observed under the blind were varied across the placebo groups, with increases rather than decreases in PLo and no significant change in PLi. Indeed, apart from placebo-induced reductions in SSRP assortativity, consistent changes across blinding conditions were not observed in other networks and graph metrics.

The data also suggest that the topological shift in SSRP configuration can continue for long periods, assuming that the blind can be maintained. In the case of PLi, this network-level adaptation was evident at 9 months with further decline in assortativity as the blind was extended an additional 12 months (see Methods). It is also noteworthy that in PLs, baseline SSRP levels correlated with improvement in motor ratings 6 months following sham surgery, whether assessed on or off dopaminergic medication. In PLi, by contrast, baseline network expression predicted the motor placebo response but only in the on-state. We note that placebo responses in PLi were generally less prominent, and may have had greater dependence on preconditioning by chronic levodopa treatment (7, 32). Under these conditions, correlations with baseline SSRP are significant only for the on-state motor ratings. We also note that the on-state placebo effect in PLi correlated with the *COMT* rs4680 polymorphism, with greater improvement in motor ratings under the blind in subjects with the Met/Met haplotype. These individuals have lower COMT enzymatic activity, which results in higher levels of prefrontal dopamine (14). It is likely that PD patients with this haplotype reach even higher cortical dopamine levels after levodopa administration.

Chronic preconditioning with dopaminergic medication appears not to be necessary, however, for the prediction of placebo responses based on pre-randomization SSRP expression. Indeed, a significant correlation was evident in the *de novo* PLo group, despite a blind phase of only 30 days. Even so, the relative reduction in SSRP assortativity under the blind in PLo (approximately 30% compared to baseline) was similar to corresponding changes in the other groups with blind phases of 6–21 months. As noted above, placebo-related decline in assortativity, a consistent finding in all three groups, was generally limited to the SSRP space. Curiously, in PLo, this graph metric was found to increase under the blind in PDRP and DMN, suggesting shift to a less stable network configuration (21, 33). The PLo group also exhibited significant placebo-related changes in other graph metrics that involved multiple network spaces as well as the whole brain (Table 1). It is not known whether the network changes seen in PLo become progressively more localized to SSRP over time, with more efficient information flow through this network as the blind is sustained.

### Structural basis for susceptibility to placebo in PD patients

In the PLo group, we used simultaneously acquired DTI to examine the relationship between baseline SSRP expression and white matter microstructure in individual subjects (see Methods). A significant cluster was found in which cingulate bundle FA correlated inversely with SSRP scores, such that the individuals with the lowest network expression had the most intact tract microstructure. Moreover, individual differences in both baseline imaging measures correlated with the motor responses to placebo under the blind. The cluster itself involved fiber tracts connecting the pregenual ACC to the NAcc. In the rodent, this pathway subserves behavioral decision making based on motivation and effort (34), whereas in the human, homologous pathways mediate self-appraisal and expectation of reward (30). We note that these regions, as well as connected downstream areas in the amygdala, thalamus, and cerebellum, are important contributors to brain circuits associated with placebo analgesia (35, 36) and placebo anxiolysis (31, 37). Whether baseline activity of these networks also correlate with corresponding placebo responses is a topic for further investigation.

### Limitations and future directions

The current data provide evidence for a consistent relationship between the SSRP and susceptibility to motor placebo effects in PD patients with symptoms of varying duration and severity. However, this study has a number of limitations. Little is known regarding its generalizability to other types of placebo responses. In PD patients, baseline expression levels of this network are in the same range as healthy volunteer subjects, have excellent test-retest reliability, and are stable in the face of disease progression and open-label levodopa treatment (9). Nonetheless, it will be useful to analyze imaging data from therapeutics trials of other disorders to determine whether analogous placebo networks can be identified. In particular, it will be critical to determine whether as with SSRP, individual placebo responses can be predicted based on pre-randomization expression levels. We additionally note that in this study, the utility of SSRP as a network marker of the placebo response was demonstrated with FDG PET, a less accessible imaging modality compared to resting-state functional MRI (rs-fMRI). Moreover, apart from its wide availability and non-invasiveness, rs-fMRI can provide single case graph metrics for correlation with clinical outcome measures in individual subjects.

The data also point to future applications of placebo networks in clinical trial design. As previously suggested, baseline SSRP expression can provide an *a priori* estimate of a subject’s susceptibility to placebo effects under blinded trial conditions (9). This approach can also be applied to subjects randomized to active treatment, in whom latent placebo effects may also contribute to the observed outcome under the blind (3). To disambiguate these responses from those associated strictly with treatment, pre-randomization measurements of SSRP expression can be used to estimate the portion of the clinical response that is attributable to placebo in a given participant. This approach offers several advantages for trial design. For example, the development of new surgical interventions for PD has been slowed by long standing ethical concerns regarding sham operations (38, 39). That said, estimation of the latent placebo response before randomization offers a practical way to reduce the need for sham procedures in PD surgical trials (11, 40). To define the best fit line relating network expression to the placebo response, one would ordinarily need to randomize 15–20 subjects to sham surgery under blinded trial conditions. Prospective validation would then be required, ideally in an independent testing set of similar size. Although additional sham surgeries may not be necessary, one can continue to randomize occasional subjects to the placebo intervention on an infrequent basis. This would allow investigators to test the stability of the prediction algorithm over time, while maintaining consistent expectation levels under the blind. An analogous strategy can be considered for pharmacological trials. Early phase blinded trials such as those described in this study (15, 41) are often underpowered and fail to meet significance criteria at the group level. In such circumstances, it may be advantageous to identify subsets of “responders,” i.e., participants with treatment responses that exceed network-based placebo estimates. This approach can be used as an adjunct to deep phenotypying for individualized treatment without necessitating modification of agreed-upon regulatory endpoints.

Finally, the SSRP can provide unique insight into the mechanisms of novel treatments. In a previous study, we reported adaptive reorganization of the pathological PDRP network in the 12 months following subthalamic AAV2-GAD gene therapy (21), manifest by increases in degree centrality and concurrent reductions in assortativity (Fig. S2A, B). In the current study, we observed analogous topological changes in the SSRP, a network in which baseline expression levels do not differ significantly in patients compared to healthy subjects (9). The persistence of the SSRP adaptation after unblinding in the gene therapy group contrasts with the reversibility of the changes in network assortativity seen with placebo (Fig. 5C, D). In aggregate, the data suggest that the reversible placebo-induced topological shift seen in SSRP may be stabilized by subthalamic gene therapy in a manner similar to what was observed for PDRP. It is likely that transfection of limbic and associative STN neurons leads to reorganization of SSRP connectivity patterns involving downstream non-motor regions (10, 17, 42). That said, the clinical benefit observed following gene therapy cannot be attributed solely to latent placebo effects mediated by SSRP. Other network mechanisms must be considered, such as reconfiguration of the PDRP (21), induction of a novel treatment-mediated network (10), or both.

## MATERIALS AND METHODS

### Subjects and study design

We analyzed data from Parkinson’s disease (PD) subjects who were randomized to placebo as part of the three independent double-blind trials (Table S1).

**1. Sham surgery:** The original study was a multicenter, randomized, double-blind sham surgery-controlled Phase 2 clinical trial to compare the effects of bilateral subthalamic adeno-associated-2 viral vector delivery of glutamic acid decarboxylase (AAV2-GAD) gene therapy to sham surgery (bilateral burr holes) over a blind phase lasting 6 months (ClinicalTrials.gov ID: NCT00643890; https://clinicaltrials.gov/study/NCT00643890) (10, 16, 17). The sham surgery group (PLs) included 21 patients with moderately advanced PD. Of these, two participants were excluded as outliers according to Tukey’s criteria, based on the observed change in Unified Parkinson’s Disease Rating Scale (UPDRS) motor ratings under the blind. Further analysis was conducted on the remaining 19 PLs subjects. All randomized subjects underwent metabolic imaging with [^18^F]-fluorodeoxyglucose positron emission tomography (FDG PET) at baseline, 6 months, and 12 months; scanning was performed at five of the participating sites as described elsewhere (10, 17). By design, the subjects in this group were simultaneously unblinded when the last participant reached 6 months from the time of surgery. In this trial, 13 of the 19 PLs subjects were unblinded before the 12-month imaging time point, while the remaining six were still under the blind when they reached the final time point. The former (unblinded) subgroup was therefore used for within-subject comparison of clinical predictions and network metrics obtained in the blinded and unblinded conditions.
**2. Injected placebo:** The second study was a single site, randomized, double-blind Phase 2 trial to compare the effects of weekly subcutaneous self-injection of exenatide (a glucagon-like peptide-1 (GLP-1) receptor agonist) or placebo (vehicle) in early levodopa-treated PD patients (ClinicalTrials.gov ID: NCT04305002; https://clinicaltrials.gov/study/NCT04305002) (41). The group randomized to injected placebo (PLi) included 29 participants who underwent FDG PET at baseline followed by repeat scanning under the blind at 9 and 21 months (i.e., after 9 additional months of weekly placebo injections, followed by a 3-month washout period during which participants in both arms remained under the blind but did not receive weekly injections). One PLi subject was excluded as an outlier with respect to the change in UPDRS motor ratings under the blind (see above), leaving 28 subjects for further analysis. All subjects were scanned using the Siemens Biograph 128 mCT PET/CT (Siemens Healthcare Molecular Imaging USA, Inc.) at Karolinska University Hospital Huddinge (Stockholm, Sweden).
**3. Oral placebo:** The third study was a single site randomized, double-blind Phase 1 trial to compare drug-naïve PD patients receiving either daily oral nicotinamide riboside (NR) or placebo under the blind for 28 days (NADPARK, ClinicalTrials.gov ID: NCT03816020; https://clinicaltrials.gov/study/NCT03816020) (15). The group randomized to oral placebo (PLo) included 15 participants who were scanned at baseline and under the blind at 28 days. All randomized subjects underwent FDG PET and simultaneous magnetic resonance imaging (MRI) on the 3T Biograph mMR MRI-PET scanner (Siemens Healthcare, Erlangen, Germany) at Haukeland University Hospital (Bergen, Norway).

Study protocols and consent forms were approved by the institutional review boards of all participating institutions. Written consent was obtained from every patient after detailed explanation of the procedures.

### Network analysis

FDG PET scans were transferred electronically to the Center for Neurosciences at The Feinstein Institutes for Medical Research (Manhasset, NY, USA) and analyzed using automated computing pipelines implemented in MATLAB 7.5 (MathWorks, Natick, MA) using in-house software (available at https://feinsteinneuroscience.org). Images were first pre-processed using Statistical Parametric Mapping (SPM) software (https://www.fil.ion.ucl.ac.uk/spm; Welcome Centre for Human Neuroimaging, London, UK). In patients in the PLs and PLi groups, who were on chronic dopaminergic treatment, FDG PET was performed in the practically defined off-state (OFF), at least 8 hours after the last dose of oral medication (10, 41). In each of the three clinical trials, metabolic scans obtained at baseline and under the blind were aligned to produce a mean image, which was spatially normalized in standard Montreal Neurological Institute (MNI) anatomic space along with the individual scans acquired at each time point. The normalized images were then smoothed with a 10-mm Gaussian filter in three dimensions to enhance the signal to noise ratio.

In this study, we utilized the previously reported SSRP topography that was identified and validated in the PLs cohort using Ordinal Trends/Canonical Variates Analysis (OrT/CVA), as described in detail elsewhere (9, 10, 13, 43). SSRP expression values for each subject at baseline and under the blind were computed on a scan-by-scan basis (software available at https://feinsteinneuroscience.org) blind to subject identity, group membership, and blinding status.

For comparison with SSRP, we additionally computed expression values for the PD-related pattern (PDRP), a distinct pathological metabolic network associated with disease progression (10, 12), as well as the metabolic default mode network (DMN) (29, 44), which may be modulated in response to placebo (30, 31, 45). Expression values for all three networks were standardized (*z*-scored) with respect to corresponding measures from 15 healthy age- and sex-matched healthy subjects (10M/5F; age 61.7 ± 2.3 years) scanned with FDG PET at the Feinstein Institutes (Manhasset, NY).

### Graph metrics

The SSRP was thresholded at z-score > 1 or <−1 and parcellated into 26 regions-of-interest (ROIs) based on the Automated Anatomical Labeling (AAL) atlas (46). At each time point, grand mean normalized regional values for each placebo group were pairwise correlated across subjects by computing Pearson product-moment correlation coefficients. To achieve graph densities of approximately 50% in each group and time point, the resulting inter-regional correlation matrices were thresholded in 0.05 increments at absolute values ranging from 0.3 to 0.6 for PLs, PLo, and healthy controls, and from 0.10 to 0.40 for PLi. The data were used to construct adjacency matrices for each group, time point, and correlation threshold from which network metrics were calculated. To enhance robustness, bootstrap resampling was performed, and the resulting graph metrics were averaged over 100 bootstrap iterations as described elsewhere (10, 21, 47).

For statistical analysis, graph metrics were computed for each group and time point across the full range of thresholds. For completeness, the total area under the curve (AUC) was used as a scalar measure of the relative magnitude of the various metrics (see below) under the various blinding conditions. For each group, differences over time and/or blinding conditions were assessed using the general linear model (GLM) with post-hoc Tukey-Kramer HSD corrections for multiple comparisons. These results were considered significant at p < 0.05 (corrected).

The following graph metrics were analyzed in each of the groups (21, 33, 47):

1. Mean degree centrality: the number of connections linking network nodes, divided by the total number of nodes that constitute the network. This measure reflects overall connectivity within the network space.
2. Assortativity coefficient: the correlation coefficient between the degree centrality for the nodes on opposite ends of a link, averaged across a given network (18, 19, 48, 49). This measure represents the propensity for nodes to form connections with nodes having similar or different attributes. As a general measure of connectional diversity in a graph, the assortativity coefficient provides an index of overall network stability (20–22).
3. Mean local clustering coefficient: the likelihood that the nearest neighbors of a node will also be connected. This metric provides an index of the degree that groups of nodes are clustered together in a given network.
4. Characteristic path length: the shortest path length between two nodes averaged over all pairs of nodes within the network space (48, 50). This metric represents the average number of hops needed to connect a given node to the others, which is inversely related to the efficiency of information flow across the entire network.
5. Small-worldness: the ratio of clustering coefficient to characteristic path length, normalized to the corresponding values from an equivalent random graph (51). This measure quantifies the ratio of regional specialization (segregation) to parallel processing (integration) of information flow along the network.

In this study, we computed changes in the graph metrics listed above for each placebo group under the blind (PLs 6 months; PLi 9 months and 21 months; PLo 28 days) with respect to the pre-randomization baseline condition. In the sham surgery group, we also compared the changes before and after unblinding in the 13 PLs subjects in whom the blind was opened before the final imaging time point (see above). In addition to SSRP, we measured the corresponding graph metric for the PDRP and DMN networks (see above) at each imaging time point. Likewise, to document the effects of placebo on whole-brain connectivity patterns, we evaluated differences in the graph metrics across conditions in each group over all 95 AAL atlas regions (33).

### Microstructural integrity and diffusion tractography

To identify localized changes in microstructural integrity associated with subject differences in baseline SSRP expression, we analyzed DTI scans acquired simultaneously with FDG PET in the baseline (prerandomization) condition of the NADPARK trial (n = 30) (15). Diffusion-weighted images were corrected for eddy current distortions and head motion using FSL (https://www.fmrib.ox.ac.uk/fsl/). Likewise, nonbrain tissue was removed from the images using the brain extraction tool in the FMRIB library. Diffusion tensor components and fractional anisotropy (FA) maps were calculated for each subject using FSL routines. We registered b0 images to the Montreal Neurological Institute (MNI-152: 2 × 2 × 2 mm^3^) template using a 12-parameter affine transformation (FLIRT) (52). The resulting transformation was then applied to the FA maps to register them to MNI space. Once aligned, the FA images were smoothed using a Gaussian kernel of 8 mm (FWHM).

Using voxel-wise multiple regression analysis in SPM 12 (https://www.fil.ion.ucl.ac.uk/spm/), we analyzed FA maps to identify white matter regions (clusters) in which FA values were significantly correlated with SSRP expression values (43). Regions were considered significant at a voxel-level threshold of p < 0.001 (uncorrected), with a cluster-level correction for multiple comparisons at p < 0.05.

For further analysis, values in significant FA clusters were grouped by quartile according to baseline SSRP expression. For validation, we made use of the DTI scans that were obtained concurrently with repeat FDG PET at 28 days in 25 of the original 30 participants (14 PLo, 11 NR; see above). To show the reproducibility of the clusters identified in the baseline scans, we compared FA values in the original cluster(s) determined at baseline with corresponding measurements from the same subjects at 28 days. This was done by computing intraclass correlation coefficients (ICC) to evaluate the test-retest reliability of the measure in the placebo and active treatment groups separately and in combination. In these two-way mixed effects models, session was treated as a fixed factor so as to isolate the variance attributable to true subject differences from residual measurement noise. In the PLo group, we correlated baseline FA values for the significant clusters with corresponding baseline SSRP values and with changes in UPDRS (motor) ratings under the blind from the same subjects. The results were considered significant for p < 0.05, Pearson product-moment correlations.

To visualize the tractographic correlates of SSRP expression in each quartile, we used DSI Studio Hou software (https://dsi-studio.labsolver.org) to reconstruct the diffusion data. This was done using generalized q-sampling imaging (53) with diffusion sampling ratio equal to 1.25. Deterministic fiber tracking was used, and augmented tracking strategies were used to improve reproducibility (54, 55). To assess relevant fiber tracks connecting SSRP nodes, regions-of-interest (ROIs) were specified in relation to the significant FA clusters using the AAL atlas (46). For fiber tracking, we used a multishell diffusion scheme: b-values of 1000 and 2500 s/mm^2^ were used, with 30 sampling directions in each shell, slice thickness = 2 mm, and in-plane resolution = 2 mm. FA values for the tract were computed and correlated post-hoc with SSRP expression. The resulting correlations were considered significant for p < 0.05.

### Statistical analysis

For statistical analysis, we correlated baseline SSRP expression with the change from baseline in UPDRS motor ratings under the blind using Pearson product-moment correlations for the PLs, PLi, and PLo groups. In each group, baseline SSRP values were correlated with changes in motor ratings assessed in the practically defined off-state (OFF) and in an on-state (ON) 1–2 hours after the subject’s usual medication dose (10, 41). To examine other variables that may influence the placebo effect, we applied multiple linear regression to the data from each cohort. Baseline SSRP expression (9) and Unified Parkinson’s Disease Rating Scale (UPDRS) motor ratings (56), as well as *COMT* rs4680 haplotype (14), were used as independent variables to model the observed changes in motor ratings (ΔUPDRS_OBS_) that took place under the blind. The best fit regression equation described above was used to estimate the latent placebo response (ΔUPDRS_EST_) in each subject, which, in turn, was compared to ΔUPDRS_OBS_ in the same individual. ΔUPDRS_OBS_ and ΔUPDRS_EST_ values were then used together to determine whether the observed response differed from the estimated placebo effect in a given individual.

As an example, we performed this analysis in the 16 PD patients who were randomized to bilateral subthalamic AAV2-GAD as part of the double-blind sham surgery-controlled RCT described elsewhere (16, 17) (https://clinicaltrials.gov/study/NCT00643890). Of these, one subject exhibited a 9.0-point increase (worsening) in UPDRS motor ratings under the blind. This far exceeded the rate of clinical progression typically seen in PD patients (57) and satisfied Tukey’s criterion for outliers (58). This subject was therefore not included in the subsequent analysis. (In the gene therapy trial, genotyping was performed in approximately half of the participants (PLs: 10/19; AAV2-GAD: 8/15). Therefore, the *COMT* rs4680 polymorphism was not included as a covariate in the regression model.) We determined whether the magnitude of the observed treatment response exceeded the estimated placebo effect on an individual case basis in both groups. Differences in the two measures were evaluated in each group using paired Student’s *t*-tests, which were considered significant for p < 0.05, two-tailed. We also computed a placebo-adjusted motor response in every subject, which was defined as the difference between ΔUPDRS_OBS_ and ΔUPDRS_EST_ in each individual. This measure was compared for the AAV2-GAD and PLs groups using Student’s *t*-test and considered significant for p < 0.05, two-tailed.

## Supplementary Material

Supplementary Files

This is a list of supplementary files associated with this preprint. Click to download.

- BarberoSupplMat.docx

## Figures and Tables

**Table 1:** Changes in Graph Metrics

Network	Cohort	Timepoint	Degree	Assortativity	Clustering	Path length	Small-worldness
**SSRP**	**PLs**	**6m**	–––	↓↓^c^	–––	–––	–––
		**12m** ^a^	–––	↑↑	–––	–––	–––
	**PLi**	**9m**	–––	↓	–––	–––	–––
		**21m** ^b^	↑↑↑	↓↓	↓↓↓	–––	↓↓↓
	**PLo**	**1m**	↑↑↑	↓↓↓	↓↓↓	↑↑↑	↓↓↓
**WB**	**PLs**	**6m**	–––	–––	–––	–––	–––
		**12m**	–––	–––	–––	–––	–––
	**PLi**	**9m**	–––	–––	–––	↑↑	–––
		**21m**	↑↑↑	–––	–––	↓↓↓	–––
	**PLo**	**1m**	↑↑↑	–––	↓↓↓	↓↓↓	↓↓↓
**PDRP**	**PLs**	**6m**	–––	–––	–––	–––	–––
		**12m**	↓↓↓	–––	↑↑↑	↓	↑↑↑
	**PLi**	**9m**	–––	–––	–––	↑↑↑	–––
		**21m**	↑	–––	↑	↓↓↓	–––
	**PLo**	**1m**	↑↑↑	↑↑↑	↓↓↓	↑↑↑	↓↓↓
**DMN**	**PLs**	**6m**	–––	↓↓↓	–––	–––	–––
		**12m**	–––	↑	↑↑↑	–––	–––
	**PLi**	**9m**	–––	–––	↑	↑	–––
		**21m**	↑	–––	↓↓	–––	–––
	**PLo**	**1m**	↑↑↑	↑↑↑	↓↓	↑↑	↓↓↓

a Comparison of the blinded (6m) and unblinded (12m) time points

b Comparison of the first (9m) and second (21m) blinded time points

c The direction of each arrow signifies the direction of change with respect to baseline. The number of arrows denotes significance: Increases: ↑ (p < 0.05), ↑↑ (p < 0.01), ↑↑↑ (0 < 0.001). Decreases: ↓ (p < 0.05), ↓↓ (p < 0.01), ↓↓↓ (p < 0.001).

## Data Availability

Deidentified data will be made available on reasonable request from interested investigators for the purpose of replicating results.
